# The rs340874 *PROX1* type 2 diabetes mellitus risk variant is associated with visceral fat accumulation and alterations in postprandial glucose and lipid metabolism

**DOI:** 10.1007/s12263-015-0454-6

**Published:** 2015-01-20

**Authors:** Adam Kretowski, Edyta Adamska, Katarzyna Maliszewska, Natalia Wawrusiewicz-Kurylonek, Anna Citko, Joanna Goscik, Witold Bauer, Juliusz Wilk, Anna Golonko, Magdalena Waszczeniuk, Danuta Lipinska, Justyna Hryniewicka, Magdalena Niemira, Magdalena Paczkowska, Michal Ciborowski, Maria Gorska

**Affiliations:** 1 Department of Endocrinology, Diabetology and Internal Medicine, Medical University of Bialystok, M.C. Sklodowskiej-Curie 24A, 15-276 Bialystok, Poland; 2 Clinical Research Centre, Medical University of Bialystok, Bialystok, Poland; 3 Centre for Experimental Medicine, Medical University of Bialystok, Bialystok, Poland; 4 Department of Dietetics and Nutrition, Medical University of Bialystok, Bialystok, Poland

**Keywords:** *PROX1* gene, Postprandial glucose/lipid metabolism, Visceral adiposity, Type 2 diabetes mellitus

## Abstract

Large-scale meta-analyses of genome-wide association studies have recently confirmed that the rs340874 single-nucleotide polymorphism in *PROX1* gene is associated with fasting glycemia and type 2 diabetes mellitus; however, the mechanism of this link was not well established. The aim of our study was to evaluate the functional/phenotypic differences related to rs340874 *PROX1* variants. The study group comprised 945 subjects of Polish origin (including 634 with BMI > 25) without previously known dysglycemia. We analyzed behavioral patterns (diet, physical activity), body fat distribution and glucose/fat metabolism after standardized meals and during the oral glucose tolerance test. We found that the carriers of the rs340874 *PROX1* CC genotype had higher nonesterified fatty acids levels after high-fat meal (*p* = 0.035) and lower glucose oxidation (*p* = 0.014) after high-carbohydrate meal in comparison with subjects with other *PROX1* genotypes. Moreover, in subjects with CC variant, we found higher accumulation of visceral fat (*p* < 0.02), but surprisingly lower daily food consumption (*p* < 0.001). We hypothesize that lipid metabolism alterations in subjects with the *PROX1* CC genotype may be a primary cause of higher glucose levels after glucose load, since the fatty acids can inhibit insulin-stimulated glucose uptake by decreasing carbohydrate oxidation. Our observations suggest that the *PROX1* variants have pleiotropic effect on disease pathways and it seem to be a very interesting goal of research on prevention of obesity and type 2 diabetes mellitus. The study may help to understand the mechanisms of visceral obesity and type 2 diabetes mellitus risk development.

## Introduction

The large meta-analyses of genome-wide association studies have confirmed that the rs340874 single-nucleotide polymorphism (SNP) in *PROX1* gene is associated with fasting glycemia and type 2 diabetes mellitus (Dupuis et al. 2010; DIAGRAM Consortium et al. 2014). *PROX1* is a transcription factor that plays a key regulatory role in neurogenesis and embryonic development of the pancreas, liver, heart and lymphatic system (Takeda and Jetten 2013; http://www.genecards.org). Tissue expression has also been found in the brain (including hypothalamic regions and hippocampus), retina, skeletal muscles, adrenal glands and gonads (http://www.genecards.org).

The link between type 2 diabetes mellitus and *PROX1* is not well established; however, the two previous studies have suggested that potential type 2 diabetes mellitus disease pathways can be related to β-cell dysfunction (Boesgaard et al. 2010; Ingelsson et al. 2010). Surprisingly, a detailed analysis of the recently published articles (Lecompte et al. 2013; Barker et al. 2011; Wagner et al. 2011) revealed that in large populations, the top hit in *PROX1* (rs340874) did not show a significant association with fasting or the oral glucose tolerance test (OGTT) insulin levels. However, there is growing evidence based on animal model studies that *PROX1* can play crucial role in the glucose/lipid metabolism in liver (Harvey et al. 2005). *PROX1* can activate transcription or function as a corepressor of wide range of genes regulating physiological processes, including HNF4α and acid-related orphan receptors (RORα and RORγ) involved in the regulation of various metabolic genes (Takeda and Jetten 2013; Jetten et al. 2013; Hayhurst et al. 2001).

The aim of our study was to analyze the functional/phenotypic associations of the rs340874 SNP in *PROX1* in humans, including the evaluation of behavioral habits (diet, physical activity), body fat distribution, insulin and nonesterified fatty acids (NEFAs) levels, as well as glucose/fat metabolism.

## Materials and methods

The study group comprised 945 (463 women and 482 men; aged 18–65 years; mean age 40.4 ± 0.8 years.) Polish origin Caucasian volunteers, without previously known dysglycemia, from the Podlasie region, recruited for *the 1000PLUS cohort* between 2009 and 2012 by the Department of Endocrinology, Diabetology and Internal Medicine, Medical University of Bialystok, Poland. Among the study population, 634 subjects were overweight/obese and 311 had BMI < 25. The study protocol was approved by the local Ethics Committee of the Medical University of Bialystok (Poland), and a written informed consent was obtained from all participants.

In all subjects, we recorded demographic and anthropometric data, collected blood samples at fasting for metabolic (glucose and insulin) and genetic analyses (*PROX1* rs340874) and performed OGTT. We conducted the 3-day food diary analysis in a randomly selected subgroup of 622 subjects. Portions of food were estimated by comparing with color photographs for each portion size (albums), as well as by asking subjects to weigh their food if possible. Daily energy, carbohydrates, fat and protein intake were analyzed using Dieta 4 software (National Food and Nutrition Institute, Warsaw, Poland). Daily physical activity was estimated using International Physical Activity Questionnaire-Long Form (IPAQ-LF), which is a self-administered questionnaire and the level of physical activity was expressed as MET (metabolic equivalent)-min per week (MET level × minutes of activity x events per week) (Hagströmer et al. 2006).

Using multi-frequency bio-impedance method—Maltron BioScan 920-2 (Maltron International Ltd, UK), we analyzed body composition: percentage of total body fat, visceral adipose tissue (VAT), subcutaneous adipose tissue (SAT) and VAT/SAT ratio.

Additionally, we performed two standardized meal tests, i.e., with high carbohydrate content and with high fat content, at an interval of 3 weeks to evaluate carbohydrate and lipid metabolism by indirect calorimetry. The tests were performed in 48 randomly selected male subjects, including 17 participants with the CC, 19 with CT and 12 with TT *PROX1* genotype (mean age 37.9 ± 1.5 years; mean BMI 28.9 ± 0.9; no significant differences between genotypes for age and BMI, *p* = 0.2 and *p* = 0.58, respectively).

### OGTT performance

We performed OGTTs according to the WHO recommendation with 75 g oral glucose dose. The participants were instructed to fast for 8–12 h prior to the tests, but not to restrict carbohydrate intake in 3 days before the test. Glycemia and insulin levels were measured at 0, 30, 60 and 120 min of the OGTT. Insulin was measured in 393 randomly selected subjects and no differences in mean age or gender composition were observed between the *PROX1* genotypes.

### Indirect calorimetry and meal tests

None of the patients enrolled in the calorimetry tests had any history of disease or treatment. They were instructed to avoid coffee, alcohol and excessive physical exercise 3 days before each test and maintain their regular lifestyle throughout the study. After an overnight—at least 12 h fast—the subjects arrived at the laboratory at 08.00 a.m. on the test day. Upon arrival at the laboratory, subjects were placed in a quiet room with thermoneutral conditions (22–25 °C) to rest for at least 30 min in a supine position (in bed). They received a standardized high-carbohydrate meal, which provided 450 kcal, including 89.3 % of energy from carbohydrate and 10.7 % of energy from protein (Nutridrink Fat Free, Nutricia Poland).

The calorimetry tests were repeated within 3 weeks with high-fat meal, which included 96 % of total energy from fat and 4 % from carbohydrates, the total kilocalories content of the meal was 450 kcal (Calogen, Nutricia Poland). No additional eating or drinking was allowed, except for small amounts (150 ml) of water during the meal tests.

Resting energy expenditure and carbohydrate and lipid oxidations were determined by computed open-circuit indirect calorimetry, measuring resting oxygen uptake and resting carbon dioxide production by a ventilated canopy (Vmax Encore 29N System, Viasys HealthCare, Yorba Linda, CA, USA) for 30 min and expressed as a 24-h value.

We evaluated fasting (0 min) and postprandial (60, 120, 180, 240 min) energy expenditure, carbohydrate and fat utilization. Moreover, at each study point, we measured glucose, insulin, NEFA and TG concentrations. Serum insulin level was measured in duplicate with the IRMA kit (Diasource ImmunoAssay S.A., Nivelles, Belgium). Plasma glucose concentration was analyzed by a hexokinase method and triglycerides by an enzymatic colorimetric method (Roche Diagnostics International Ltd, Switzerland). Serum NEFA were quantified using a kit from Zen-Bio, Inc. (Research Triangle Park, NC, USA).

In order to evaluate insulin resistance, we used the homeostasis model assessment: HOMA-IR = fasting insulin (µU/ml) × fasting glucose (mmol/l)/22.5 and β-cell function: HOMA-B = [insulin (µU/ml) × 20]/[glucose (mmol/L)—3.5] (Matthews et al. 1985). Corrected insulin response at 30 min of OGTT (CIR 30 min) was calculated according to Sluiter et al. (1976) as: ([serum insulin_30min_ (pmol/l)/6.45] × 100)/(plasma glucose_30min_(mmol) × [plasma glucose_30min_ (mmol/l)—3.89]) to evaluate early phase of insulin secretion.

### Genetic analyses

DNA was extracted from the peripheral blood leukocytes using a classical salting out method. All SNPs were genotyped by TaqMan SNP technology from ready to use human assays library (Applied Biosystems, USA) using a high throughput genotyping system—OpenArray from Life Technologies (USA). SNPs analysis was performed in duplicate, following the manufacturer’s instructions. As a negative control, we used a sample without template. The negative control was helpful in measuring any false positive signal caused by contamination. No significant deviation from Hardy–Weinberg equilibrium was observed for the studied rs340874 SNP in *PROX1* (*p* > 0.05).

### Statistical analyses

To assess statistically significant differences between groups defined by genotypes with one of the quantitative variables set as a response variable either fitting a linear model for each stratum or Kruskal–Wallis test was performed. The choice was made upon meeting the normality and homogeneity of variance assumptions. When the test showed to be significant, post hoc analysis was performed with the use of pairwise *t* test or Wilcoxon test, and due to the problem of multiple testing (multiple pairwise comparisons), false discovery rate (FDR) *p* value adjustment was used (Benjamini and Hochberg 1995). The R software environment was employed for all calculations (R Core Team 2012). Multivariable generalized linear model with a binomial link function was also used for the analysis of the association of the risk genotype with the study traits.

## Results

Among 945 subjects enrolled in our study, 67 cases of type 2 diabetes mellitus have been diagnosed based on fasting glycemia ≥126 mg/dl or 2 h glycemia ≥200 mg/dl at OGTT. In 646 participants, fasting and glucose levels during the OGTT were normal. There was a significant difference (*p* = 0.039) in distribution of the studied *PROX1* C allele (*χ* ^2^ = 4.26) between the subjects with normal glucose levels and those with type 2 diabetes mellitus—48.8 versus 58.2 %, respectively. Moreover, in the logistic regression model, the *PROX1* CC genotype was associated with type 2 diabetes mellitus risk [OR 2.8 (1.1–8.7), *p* = 0.044] when adjusted for age, sex, BMI, HOMA-IR and VAT/SAT ratio (recessive model).

Demographic, anthropometric and laboratory data at fasting state by the rs340874 *PROX1* genotypes are presented in Table 1. The *PROX1* CC genotype carriers were more likely to have increased visceral fat tissue content (CC vs. CT vs. TT: 114.4 vs. 96.1 vs. 94.0 cm^3^, respectively, *p* = 0.019). When the multivariate generalized linear model was used, the *PROX1* CC genotype was associated with VAT/SAT ratio [OR 1.5 (1.01–2.3), *p* = 0.041] adjusted for age, gender, BMI, fasting insulin, diet and/or treatment of obesity. However, HOMA-IR was not associated with the *PROX1* CC genotype (data not shown) in similar GLM model.

**Table 1:** Association of rs340874 *PROX1* genotypes with demographic, anthropometric, behavioral (food intake and physical activity) data and laboratory measurements at fasting state

Parameter	CC	CT	TT
*N*	246	442	257
Age (years)	42.6 ± 0.9	39.3 ± 0.7	40.0 ± 0.9
Gender (% male)	48.8	48.9	55.6
BMI	29.0 ± 0.5	28.0 ± 0.3	28.1 ± 0.4
WHR	0.93 ± 0.005	0.93 ± 0.004	0.93 ± 0.005
Fat content (%)	30.7 ± 0.8	29.1 ± 0.6	28.5 ± 0.8
VAT (cm^3^)*	114.4 ± 6.8	96.1 ± 4.0	94.1 ± 5.4
VAT/SAT	0.70 ± 0.04	0.63 ± 0.02	0.62 ± 0.03
Insulin fasting (IU/ml)	13.8 ± 1.0	12.5 ± 0.5	12.5 ± 0.7
Fasting glucose (mg/dl)	98.5 ± 0.7	96.5 ± 1.6	95.3 ± 1.0
HOMA-B	163.0 ± 13.3	163.9 ± 8.5	149.3 ± 16.3
HOMA-IR	3.4 ± 0.3	3.1 ± 0.1	3.1 ± 0.2
Total energy intake (kcal/day)^†^	1,736 ± 54	1,846 ± 51	2,053 ± 67
Carbohydrate intake (g/day)^‡^	223 ± 6	232 ± 6	262 ± 9
Protein intake (g/day)	80 ± 2^§^	85 ± 2	94 ± 3
Fat intake (g/day)	61 ± 3	66 ± 3	70 ± 3
Food energy density	0.86 ± 0.03	0.87 ± 0.02	0.89 ± 0.03
Physical activity (MET)	10,056 ± 562	9,616 ± 394	10,422 ± 568

Values are mean ± SE, unless otherwise indicated

Significant *p* values are presented: * *p* < 0.02, ^†^ *p* < 0.001, ^‡^ *p* < 0.002, ^§^ *p* < 0.01

The association of rs340874 *PROX1* genotypes with total energy intake, macronutrient content based on 3 days nutritional self-reports and physical activity is presented in Table 1. Carriers of *PROX1* two C alleles presented significantly lower total energy, carbohydrate and protein daily intake. There was also a trend for lower daily fat intake (*p* = 0.15). These differences were still observed when the analyses had been repeated in subjects who did not declare any dietary restrictions (data not shown). There were no differences in physical activity related to *PROX1* genotypes in the studied population.

Glucose and insulin levels during the OGTT by *PROX1* rs340874 genotypes are presented in Table 2. Since the results of the OGTT test for diagnosis of type 2 diabetes mellitus are reliable when the test is done in subjects without calories restriction and without any concomitant illnesses and/or treatment, which might affect the results, such as clinically significant endocrine, renal, hepatic and gastrointestinal disorders, subjects who did not fulfill the above criteria were not included in further analysis. In the remaining 734 participants, we observed significantly higher glucose levels for the *PROX1* CC genotypes in comparison with subjects with CT or TT variants at 30 min (*p* < 0.05), 60 min (*p* = 0.023) and 120 min (*p* < 0.05) of the OGTT (Table 2).

**Table 2:** Glucose and insulin levels during the OGTT by rs340874 *PROX1* genotypes

Parameter	CC	CT	TT
Glycemia (mg/dl)			
*N*	179	343	212
0 min	98 ± 2.0	96 ± 1.1	95 ± 1.3
30 min*	152 ± 4.7	140 ± 2.0	141 ± 2.5
60 min^†^	132 ± 4.1	122 ± 2.7	123 ± .2
120 min*	102 ± 3.4	95 ± 2.0	93 ± 2.1
Insulin (IU/ml)			
*N*	110	171	112
0 min	12.9 ± 1.3	11.0 ± 0.5	12.3 ± 0.8
30 min	73.0 ± 4.4	81.7 ± 3.9	75.5 ± 4.8
60 min	78.6 ± 5.5	76.3 ± 4.6	88.0 ± 9.5
120 min	47.8 ± 5.1	39.5 ± 2.7	50.4 ± 4.4
CIR_insulin30_^‡^	0.81 ± 0.06	1.0 ± 0.07	0.92 ± 0.08
AUC	7,339 ± 482	7,291 ± 356	7,947 ± 619

* *p* < 0.05, ^†^ *p* = 0.023, ^‡^ *p* = 0.07

**Fig. 1:**
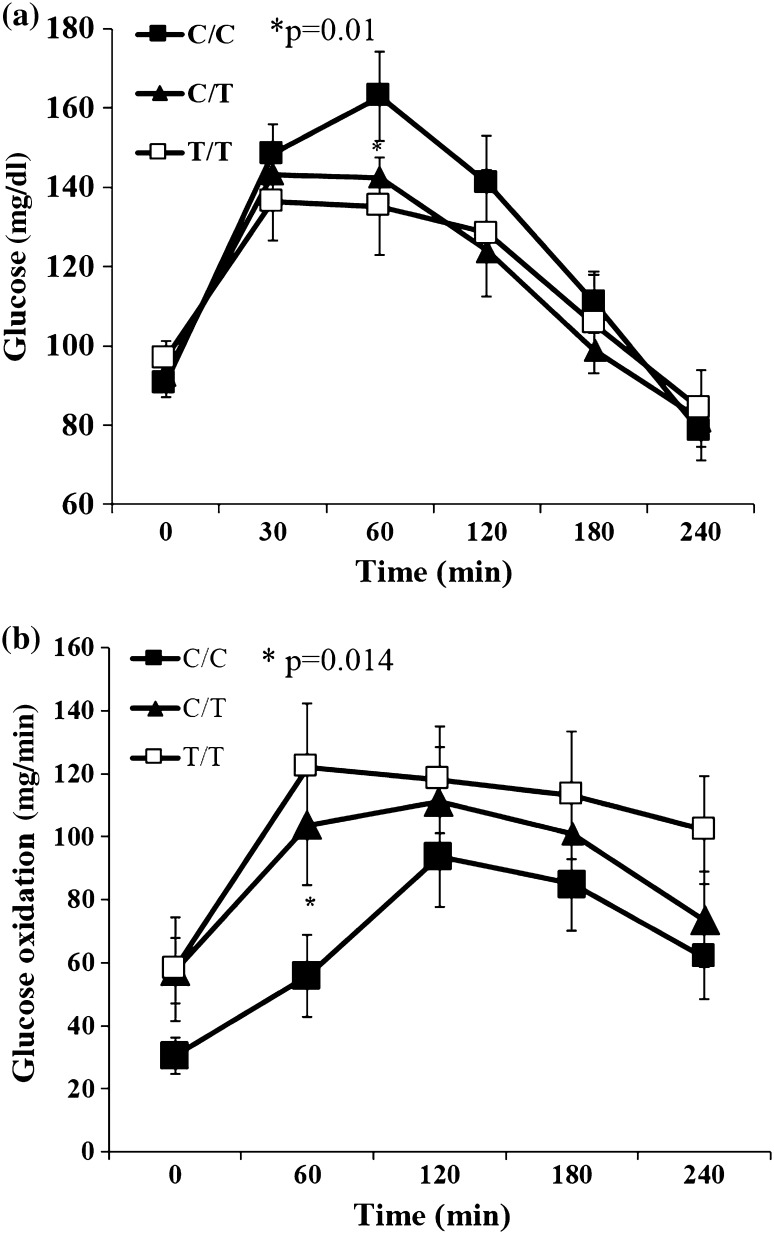
Glucose levels (**a**) and glucose oxidation (**b**) after high-carbohydrate meal intake by the rs340874 *PROX1* genotype

No significant differences of insulin levels and insulin AUC during OGTT between the studied *PROX1* genotypes were observed (Table 2). There was only a tendency for the lower corrected (by glucose levels) insulin release at 30 min of OGTT (CIR_insulin30_) for the CC genotype vs. other *PROX1* genotypes, 0.81 ± 0.06 versus 1.0 ± 0.07 versus 0.92 ± 0.08, respectively (*p* = 0.07). Similarly, higher glucose levels were observed in subjects with the *PROX1* CC genotypes at 60 min during high-carbohydrate test meal (CC vs. TT: 162.9 ± 11.3 vs. 135.2 ± 12.2 mg/dl, *p* = 0.015) (Fig. 1a).

**Fig. 2:**
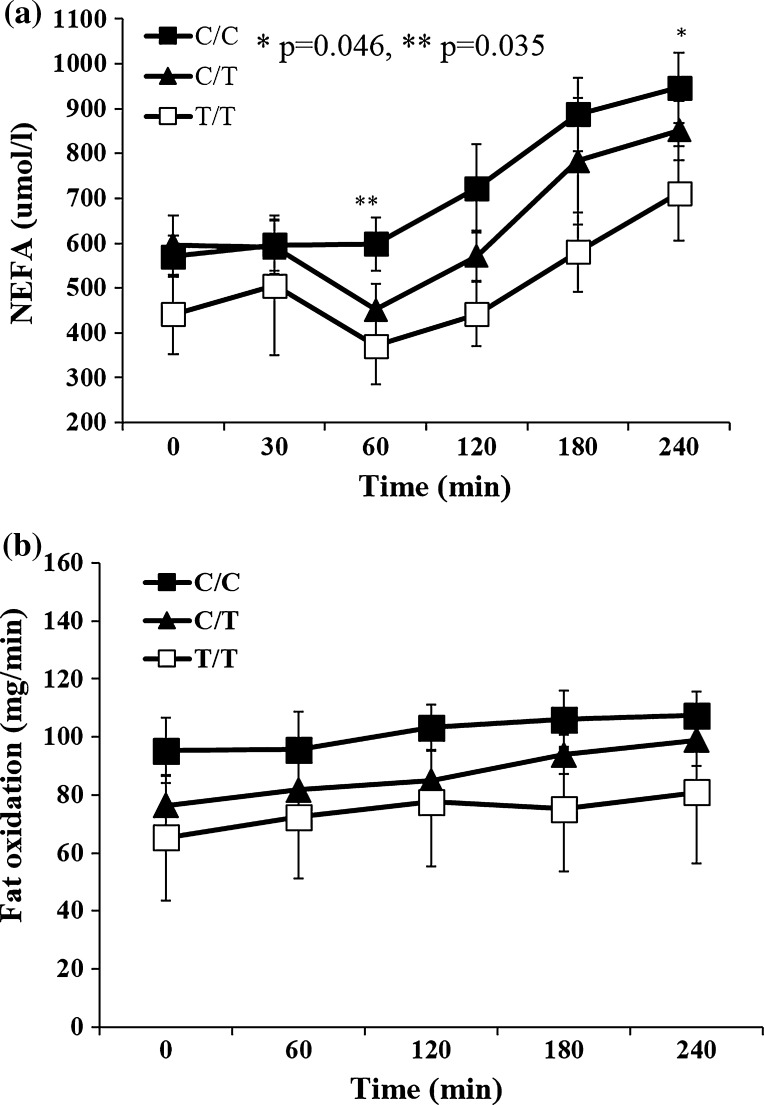
NEFA levels (**a**) and fat oxidation (**b**) after high-fat meal intake by the rs340874 *PROX1* genotype

The carriers of the *PROX1* CC variant had higher nonesterified fatty acid levels during standard high-fat meal—for CC versus CT + TT at 60 min 597.8 ± 58.9 versus 430.5 ± 47.5 μmol/l, *p* = 0.035 and at 240 min 946.7 ± 78.9 versus 748.6 ± 56.3 μmol/l, *p* = 0.046 (Fig. 2a). Additionally, there was a trend for higher TG levels at fasting for CC versus CT + TT genotypes TG_0 min_: 118.5 ± 18.7 versus 87.0 ± 8.2, *p* = 0.11; the difference became significant at 60 and 180 min after high-fat meal, i.e., TG_60min_ 127.1 ± 23.7 versus 82.8.0 ± 8.2, *p* = 0.05 and TG_180min_ 205.7 ± 28.0 versus 145.2.0 ± 12.5, *p* = 0.044.

### Glucose/fat oxidation and energy expenditure

In the subjects with *PROX1* CC genotype, indirect calorimetry analyses revealed significantly lower glucose oxidation at 60 min after high-carbohydrate meal, CC versus TT: 55.8 ± 12.9 versus 122.1 ± 20.3 mg/min, *p* = 0.014 (Fig. 1b). There were no statistical differences in the fat oxidation at fasting and during high-fat meal (Fig. 2b) associated with *PROX1* genotypes—for CC versus CT versus TT AUC for fat oxidation was 4.4 ± 0.2 versus 4.8 ± 0.1 versus 6.8 ± 2.3 mg/min/FFM, respectively (*p* = 0.34). Finally, there was a tendency for lower energy expenditure for CC genotype versus other genotypes during high-carbohydrate meal (for CC vs. CT + TT: EE_AUC_HC_ = 4.6 ± 0.1 vs. 4.9 ± 0.1, *p* = 0.09) and during high-fat meal (EE_AUC_HF_ for CC vs. CT + TT: 4.4 ± 0.2 vs. 5.3 ± 0.6 kcal/min/FFM *p* = 0.18).

## Discussion

In the present study, we showed for the first time that the association of the rs340874 C allele with type 2 diabetes mellitus can be related to the NEFA/glucose metabolism alterations. We found that the carriers of the rs340874 in *PROX1* CC genotype had higher NEFA levels after high-fat meal and lower glucose oxidation in comparison with subjects with other *PROX1* genotypes. Since the fatty acids can inhibit insulin-stimulated glucose uptake by decreasing carbohydrate oxidation and glycogen synthesis (Boden et al. 1994), it seems highly probable that lipid alterations may be a primary cause of higher glucose levels after glucose load and during high-carbohydrate meal in subjects with the *PROX1* CC genotype. Interestingly, there were no differences in the BMI among subjects with different *PROX1* genotype, but the higher accumulation of visceral fat was found in the *PROX1* CC risk group.

We did not observe any statistically significant differences in fasting and the OGTT insulin levels by the studied *PROX1* genotypes. Similarly, no association of the rs340874 SNP with fasting or 2 h OGTT insulin was found in the large meta-analysis comprising more than 120 K individuals, while there was a highly significant effect of the studied *PROX1* variant on fasting glucose and type 2 diabetes mellitus risk (Dupuis et al. 2010).

Our findings are also in line with the other meta-analysis by Ingelsson et al. (2010), comprising more than 29,000 of subjects. The authors did not find any effect of the rs340874 SNP in *PROX1* on insulin sensitivity and proinsulin levels. In fact, this large population showed a tendency for higher proinsulin levels in subjects with prodiabetogenic C allele (*β*_adjusted_ for age + sex = 0.0069 + 0.005, *p* = 0.18).

These observations are also confirmed by the data from the group of 1,782 German subjects at increased risk of type 2 diabetes mellitus who underwent OGTT (Wagner et al. 2011). The AUC of proinsulin/insulin ratio during 60–120 min of OGTT was significantly higher among CC genotype carriers. Wagner et al. (2011) found higher fasting glucose and lower insulin/glucose ratio (AUC_insulin0–30_/AUC_glucose0-30_) but not with insulin levels. These findings could indicate that elevated proinsulin and proinsulin/insulin ratios are secondary to increased demands on β-cell secretion induced by hyperglycemia and/or insulin resistance, as shown by Birkeland et al. (1994).

Similarly to our study, no associations of the rs340874 SNP in *PROX1* with fasting insulin, HOMA-B and HOMA-IR were observed in a group of 1,155 adolescents (12–18 years of age) in the study by Lecompte et al. (2013). However, in their more complex analysis of *PROX1* genetic variability (80 SNPs), other SNPs (rs340838, rs340837, rs340836) were found to be significantly associated with lower fasting insulin levels, lower HOMA-B and surprisingly with lower HOMA-IR. Further analyses of *PROX1* SNPs impact on luciferase activity using transfection assays have shown the association of rs340873 A allele and rs340835 A variants with lower expression in both the pancreas and liver. On the other hand, the rs340874 *PROX1* C allele was associated with a 1.65-fold lower luciferase activity in HepG2 hepatic cells only. Finally, Lecompte et al. (2013) suggest that in fact the rs340874 SNP in *PROX1* seems to be the functional variant in the liver since it shows the differences in allele binding with a 60 % higher affinity for the C allele than for the T allele at electrophoretic mobility shift assay.

Based on the previously published studies and our current data, we can conclude that there is a pleiotropic mechanism of *PROX1* gene in different tissues related to different allelic variants. This can be confirmed by the fact that the rs340874 SNP is only in a modest LD (*r* ^2^ ~ 0.6) with other SNPs, that have the effect on insulin-related traits.

Surprisingly, in the group with the *PROX1* CC genotype and a higher risk of type 2 diabetes mellitus, we found significantly lower food consumption, but higher accumulation of visceral fat. This was also observed in the subgroup of patients who did not report any intentions regarding possible efforts to lose weight or calories restriction (data not shown). Our present observations are, however, in line with the recent experiments on animal models. In the study by Harvey et al. (2005), the *PROX1* ± adult mice (with *PROX1* haploinsufficiency) presented intra-abdominal fat accumulation, increased deposition of liver lipids, but also a tendency to lower food consumption after the onset of obesity. In this very elegant experiment, the authors did not observe any significant differences in the expression of hypothalamic proteins that control appetite between *PROX1* ± mice and their wild-type counterparts (Harvey et al. 2005). We can only speculate that the changes in food intake can be related to the function/pathology of hippocampus since the *PROX1* overexpression is associated with neuronal differentiation within the hippocampal niche (Karalay et al. 2011). There is also growing evidence suggesting that dietary factors are associated with the emergence of hippocampal pathology and that hippocampal pathology is associated with the emergence of food intake and body weight gain (Kanoski and Davidson 2011).

In conclusion, even if our study has some limitations as using of bioimpendance method to measure body fat distribution, the results suggest that *PROX1* variant has a pleiotropic effect on type 2 diabetes mellitus risk, and the rs340874 C/T *PROX1* SNP is associated with NEFA/glucose metabolism alterations rather than defects of insulin secretion. We believe that the study may help to understand the mechanisms of visceral obesity and type 2 diabetes mellitus risk development. Finally, it seems that the *PROX1*-related pathways are the very interesting goal of research on prevention of obesity and type 2 diabetes mellitus.
